# Androgenic suppression combined with radiotherapy for the treatment of prostate adenocarcinoma: a systematic review

**DOI:** 10.1186/1471-2407-12-54

**Published:** 2012-02-02

**Authors:** André D Sasse, Elisa Sasse, Albertina M Carvalho, Ligia T Macedo

**Affiliations:** 1 Center for Evidences in Oncology, Clinical Oncology Service, Internal Medicine Department Faculty of Medical Sciences, University of Campinas--UNICAMP, 6111, 13083-970 Campinas, SP, Brazil; 2 Medical School, Federal University of Rio de Janeiro (UFRJ), Rio de Janeiro, Brazil; 3 Oncology Center (Centro Oncologico), Luanda, Angola

## Abstract

**Background:**

Locally advanced prostate cancer is often associated with elevated recurrence rates. Despite the modest response observed, external-beam radiotherapy has been the preferred treatment for this condition. More recent evidence from randomised trials has demonstrated clinical benefit with the combined use of androgen suppression in such cases. The aim of this meta-analysis is to compare the combination of distinct hormone therapy modalities versus radiotherapy alone for overall survival, disease-free survival and toxicity.

**Methods:**

Databases (MEDLINE, EMBASE, LILACS, Cochrane databases and ClinicalTrials.gov) were scanned for randomised clinical trials involving radiotherapy with or without androgen suppression in local prostate cancer. The search strategy included articles published until October 2011. The studies were examined and the data of interest were plotted for meta-analysis. Survival outcomes were reported as a hazard ratio with corresponding 95% confidence intervals.

**Results:**

Data from ten trials published from 1988 to 2011 were included, comprising 6555 patients. There was a statistically significant advantage to the use of androgen suppression, in terms of both overall survival and disease free survival, when compared to radiotherapy alone. The use of long-term goserelin (up to three years) was the strategy providing the higher magnitude of clinical benefit. In contrast to goserelin, there were no trials evaluating the use of other luteinizing hormone-releasing hormone (LHRH) analogues as monotherapy. Complete hormonal blockade was not shown to be superior to goserelin monotherapy.

**Conclusions:**

Based on the findings of this systematic review, the evidence supports the use of androgen suppression with goserelin monotherapy as the standard treatment for patients with prostate cancer treated with radiotherapy, which are at high risk of recurrence or metastases.

## Background

Prostate cancer is the second most prevalent neoplasm and the sixth leading cause of cancer mortality, with an estimated incidence of 903,500 new cases and 258,400 deaths in 2008 [1]. Historically, the incidence of this condition had a rapid increase during the early 1990s with later stabilization. This can be largely explained by the introduction of prostate specific antigen (PSA) testing, causing a sudden diagnosis boost worldwide. For the same reason, the distribution of prostate cancer cases was heterogeneous, with higher rates observed in the United States, Australia, Norway, Japan, Italy and others [2]. On the other hand, mortality has been declining in such countries, probably as a consequence of recent advances in treatment and support [3].

The advent of improved curative intent therapy for local disease has been suggested as one of the reasons for the decrease of prostate cancer deaths in the past decade. Current strategies for the management of localized prostate cancer consist of either surgical treatment or radiotherapy. Until the present, there has been no accurate comparison coming from randomized clinical trials evaluating the clinical efficacy of these strategies. Another reason could be related to response to therapy, which is highly dependent on other prognostic factors. For example, patients classified as low-risk (PSA ≤ 10 ng/ml, Gleason ≤ 6, stage T1c or T2a) and treated with radiotherapy have better prognosis, with a reported 10 year mortality of 2%, while higher risk patients face mortality rates ranging from 12% to 30% [4]. On the other hand, a surgical approach for locally advanced prostate cancer had shown cure rates of less than 25% [5]. Therefore, external-beam radiotherapy is usually the preferred treatment in this setting, due to an acceptable survival rate [6] and less morbidity than surgery. Nonetheless, patients with locally advanced disease have the worst prognosis and higher chances of relapse [7].

Since the 1970s, the role of androgen deprivation therapy adjuvant to radiotherapy to treat locally advanced prostate cancer has been investigated [8]. The inhibitory effect of androgen deprivation on the growth and proliferation of prostate cancer cells is well established. Several studies have shown a rapid regression of prostate cancer after total androgen blockade, leading to hypothesize that the marked regression observed at the prostatic level of both malignant and non-malignant tissue may increase radiation efficacy. In decreasing the tumor size by antiandrogen medication, an optimal dose of radiation could treat adequately prostate cancer with less adverse events.

In this context, androgen deprivation therapy has been a matter of discussion and study as a complementary treatment for high-risk patients. Evidence in favour of its use combined with radiotherapy has been described, with benefit in terms of overall survival [9]. However, the optimal time for introduction, duration of hormone therapy, and the type of suppression involved are still unanswered issues.

In this meta-analysis, the authors intended to summarize all the published data to perform appropriate comparisons between the different regimens used for androgen deprivation in patients receiving radiotherapy for localized or locally advanced prostate cancer.

## Methods

### Search strategy

Search strategies were performed in relevant electronic databases, including PubMed/MEDLINE, EMBASE, LILACS, ClinicalTrials.gov and The Cochrane Library for randomized studies evaluating radiotherapy with or without androgen suppression in patients with localized or locally advanced prostate cancer. Articles published or presented from January 1966 to October 2011 were identified. Details of the search strategy used for PubMed/MEDLINE are described separately (Table 1).

**Table 1:** Description of search terms used (for MEDLINE database)

#1 "goserelin acetate"	#55 Prostacur
#2 goserelin	#56 Prostica
#3 "ICI-118630"	#57 SCH-13521
#4 "ICI 118630"	#58 "SCH 13521"
#5 "ICI118630"	#59 "SCH13521"
#6 Zoladex	#60 Prostogenat
#7 #1 OR #2 OR #3 OR #4 OR #5 OR #6	#61 Testotard
#8 Leuprorelin	#62 Apimid
#9 Enantone	#63 #34 OR #35 OR #36 OR #37 OR #38 OR #39 OR #40
#10 "Leuprolide Acetate"	OR #41 OR #42 OR #43
#11 "Leuprolide Monoacetate"	OR #44 OR #45 OR #46 OR #47 OR #48 OR #49 OR
#12 Lupron	#50 OR #51 OR #52
#13 "TAP-144"	OR #53 OR #54 OR #55 OR #56 OR #57OR #58 OR
#14 "TAP 144"	#59 OR #60 OR #61
#15 "TAP144"	OR #62
#16 "A-43818"	#64 Receptal
#17 "A 43818"	#65 Buserelin
#18 "A43818"	#66 Bigonist
#19 #8 OR #9 OR #10 OR #11 OR #12 OR #13 OR #14	#67 Tiloryth
OR #15 OR #16 OR #17	#68 Profact
OR #18	#69 Suprecur
#20 diethylstilbestrol	#70 Suprefact
#21 "Stilbene Estrogen"	#71 "HOE-766"
#22 Stilbestrol	#72 "HOE 766"
#23 Apstil	#73 "HOE766"
#24 Tampovagan	#74 #64 OR #65 OR #66 OR #67 OR #68 OR #69 OR #70
#25 Distilbène	OR #71 OR #72 OR #73
#26 Agostilben	#75 cyproterone
#27 #20 OR #21 OR #22 OR #23 OR #24 OR #25 OR #26	#76 Androcur
#28 "ICI-176334"	#77 Cyprone
#29 "ICI 176334"	#78 Cyprostat
#30 Casodex	#79 "SH 714"
#31 bicalutamide	#80 "SH-714"
#32 Cosudex	#81 "SH714"
#33 #28 OR #29 OR #30 OR #31 OR #32	#82 #75 OR #76 OR #77 OR #78 OR #79 OR #80 OR #81
#34 Niftolid*	#83 Orchiectom*
#35 flutamide	#84 Castration*
#36 Chimax	#85 #83 OR #84
#37 Cytamid	#86 prostate
#38 Eulexin*	#87 prostatic
#39 Drogenil	#88 #86 OR #87
#40 Euflex	#89 neoplasm*
#41 Fluken	#90 Cancer*
#42 Flulem	#91 Tumor*
#43 Flumid	#92 Malignan*
#44 Fluta 1A Pharma	#93 #89 OR #90 OR #91 OR #92
#45 "Fluta-cell"	#94 radiotherap*
#46 "Fluta cell"	#95 "external beam radiation"
#47 Flutacell	#96 radiation
#48 Flutamin	#97 brachitherapy
#49 Flutandrona	#98 #94 OR #95 OR #96 OR #97
#50 Flutaplex	#99 random*
#51 Flutexin	#100 #7 AND #19 AND #27 AND #33 AND #63 AND #74
#52 Fugerel	AND #82 AND #85 AND
#53 Grisetin	#88 AND #93 AND #98 AND #99
#54 Oncosal	

### Selection criteria

The purpose of this study was to identify all published randomised, controlled clinical trials in English, Spanish or Portuguese, comparing radiotherapy with or without any androgen suppression (orchiectomy, luteinizing hormone-releasing hormone [LHRH] analogues, peripheral anti-androgens or estrogenic therapy) in localized (cT1-2) or locally advanced (T3-4 N0-2 M0) prostate carcinoma. We included studies that evaluated introduction of androgen suppression either before, during or after radiotherapy; and excluded those for which anti-androgenic treatment was performed in both arms. Two researchers (ES, AMC) independently examined the list of references, as well as article selection.

### Data extraction

For identification purposes, the selected trials were named after their first author and year of publication. Overall survival (OS), disease-free survival (DFS) and toxicity were the main outcomes of interest. For continuous variables of survival, we looked for the original hazard ratios (HR). Whenever data reported were unavailable or incomplete for analysis, we attempted to estimate it by applying either the described number of events and the corresponding p-value for the log-rank statistic, or by transcription of survival curves as suggested by Parmar and colleagues [10]. In this case, calculations were then made through a spreadsheet developed by Tierney and colleagues [11].

### Statistical analysis and synthesis

Evaluation of possible bias, as described by Sterne and colleagues [12] was performed by two authors (ADS, ES). Special focus was given to the availability of information about randomization, blinding, allocation concealment, description of dropouts, utilization of an intention-to-treat (ITT) analysis, and source of funding.

Meta-analyses for this study were conducted with RevMan 5.0 software (Cochrane Collaboration's Information Management System). Analyses of data consisted of the HR for time-to-event outcomes and odds ratio (OR) for dichotomous variables (adverse events) for which 95% confidence intervals (CI) were calculated and presented in forest plots. The diamond at the bottom of the plot summarizes the best estimate results (the width representing its corresponding 95% CI). Statistical heterogeneity was evaluated with the chi-square test [13], and expressed using the *I^2 ^*index, as described by Higgins and colleagues [14]. If heterogeneity was detected (*I^2 ^*> 50%), a possible explanation was investigated. The presence of possible publication bias regarding the first endpoint (OS) was investigated by a funnel plot.

## Results

### Search results

**Figure 1:**
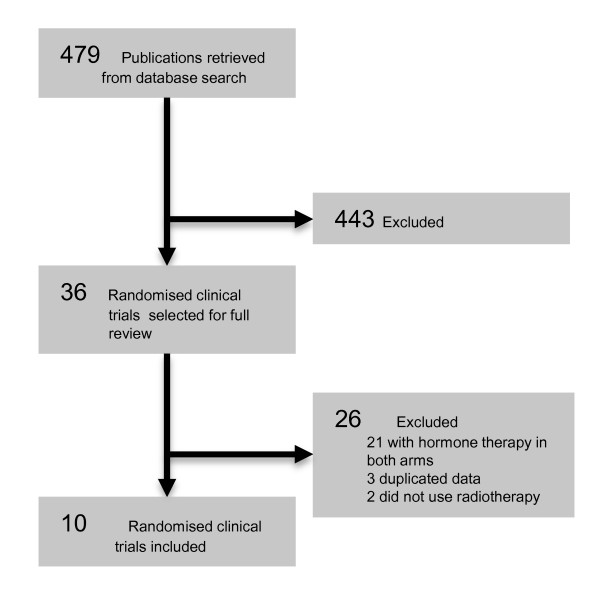
**Selection Results**. QUOROM flowchart of the systematic literature review.

From 479 potential studies identified with the search strategies applied, ten published articles from 1988 to 2011 were included, comprising 6555 enrolled patients. The literature search results are summarized in Figure 1.

Concerning the methods of androgenic deprivation therapy evaluated in these studies, three trials involved central blockade (through goserelin [15,16] or orchiectomy [17]), while four studied combined suppression with flutamide [18-22], one used peripheral blockade with bicalutamide [23], and another study reported the use of estrogenic treatment [8]. Further details on study design and treatment modalities are described in Tables 2 and 3, respectively.

**Table 2:** Methodological characteristics of clinical trials

Author	Year	Rand.	Alloc.	Blind.	Desc. losses	Sample Size	ITT	Multic.	Sponsor
Zagars	1988	NS	NS	No	Yes	No	No	No	Public

Laverdiere	2004	NS	NS	No	No	No	Ns	No	NS

Lawton	2005	Yes	Yes	No	Yes	Yes	No	Yes	NS

Granfors	2006	NS	NS	No	Yes	Yes	Yes	No	Public

See	2006	Yes	Yes	Yes	Yes	Yes	Yes	Yes	Industry

D'Amico	2008	Yes	Yes	No	Yes	Yes	Yes	Yes	Public

Roach	2008	NS	NS	No	Yes	Yes	Yes	Yes	Public

Bolla	2010	Yes	Yes	No	Yes	Yes	Yes	Yes	Both

Denham	2011	Yes	Yes	No	Yes	Yes	No	No	Both

Jones	2011	Yes	Yes	No	Yes	Yes	Yes	Yes	Public

Abbreviations: *Alloc *allocation concealment, *Blind *blinding, *Desc. Losses *description of losses to follow-up, *ITT *intention-to-treat analysis, *Multic *multicentric, *NS *not stated, *Rand *adequate randomization method, *Sample Size *adequate planning and calculation of sample size

**Table 3:** Designed therapies

Author	Year	Radiotherapy (dose)	Hormone Therapy	Duration	N	Median follow up
Zagars	1988	70 Gy	Diethylstilbestrol 25 mg PO qd	Continuously	82	14.5 years

Laverdiere	2004	64 Gy	Leuprolide 7.5 mg/month + Flutamide	3 months or 10 months	161	5 years

Lawton	2005	65 to 70 Gy	Goserelin 3.6 mg/month	Continuously	977	6.5 years

Granfors	2006	60 to 70 Gy	Orchiectomy	Permanent	91	9.7 years

See	2006	NS	Bicalutamide 150 mg PO qd	Decided by investigator	1370	7.2 years

D'Amico	2008	NS	Goserelin 3,6 mg **or** Leuprolide 7.5 mg/month + Flutamide	6 months	206	8.2 years

Roach	2008	65 to 70 Gy	Goserelin 3.6 mg/month + Flutamide	3 months	456	11.9 years

Bolla	2010	70 Gy	Goserelin 3.6 mg/month	3 years	415	9.1 years

Denham	2011	66 Gy	Goserelin 3.6 mg/month + Flutamide	3 months or 6 months	818	10.6 years

Jones	2011	66.6 Gy	Goserelin 3,6 mg or Leuprolide 7.5 mg/month + Flutamide	4 months	1979	9.1 years

Abbreviations: *Gy *Gray unit, *N *number of patients, *NS *not stated, *PO *oral administration, *qd *every day

### Overall survival

Data regarding OS was available in nine trials. Only four presented statistically significant results in favour of androgen deprivation. The funnel plot analysis performed confirmed the absence of publication bias. Due to the high overall heterogeneity observed between trials, we conducted subgroup analyses for more homogeneous comparisons.

#### Overall survival per androgen suppression modality

**Figure 2:**
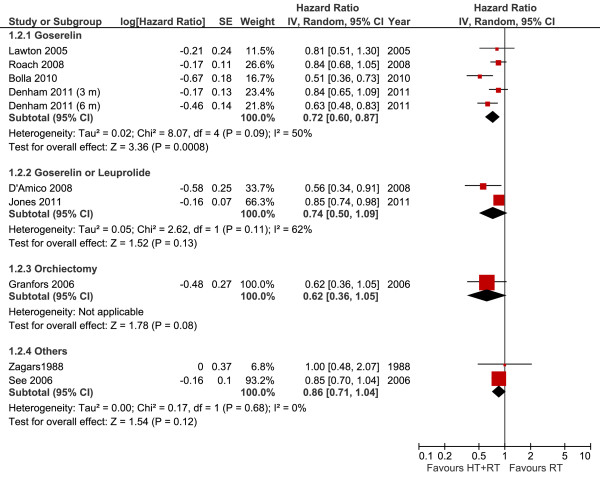
**Overall Survival per Androgen Suppression Meta-analysis**. Meta-analysis of overall survival, comparing hormone therapy combined with radiotherapy versus radiotherapy alone in patients with non-metastatic prostate cancer (Note: In the study by D'Amico, most patients received Leuprolide). Abbreviations: CI = confidence interval; HT--hormone therapy; IV = Generic Inverse Variance; RT--radiotherapy; SE = standard error.

The use of goserelin combined with radiotherapy was associated with a 28% reduction of the risk of death (OS results: HR 0.72, 95% CI 0.60-0.87, *P *= 0.0008) (Figure 2). Although some heterogeneity was noted (*I^2 ^*= 50%), the advantage of hormone therapy was present in all studies. A probable cause for this could be the diverse treatment durations leading to distinct clinical efficacy data.

In two trials [20,21], either goserelin or leuprolide was combined with flutamide. The high heterogeneity (*I^2 ^*= 62%) precludes the interpretation of the meta-analysis, and can be explained by the different inclusion criteria from the trials.

In case of orchiectomy, one study showed the same pattern of advantage, yet failed to demonstrate statistical significance of the benefit (HR 0.62, 95% CI 0.36-1.05, *P *= 0.08). This finding may be explained by the small number of patients included in the analysis.

The use of bicalutamide or estrogenic therapy without LHRH analogues did not result in evidence of OS benefit (HR 0.85, 95% CI 0.70-1.04, and HR 1.00, 95% CI 0.48-2.07, respectively).

#### Overall survival according to hormonal blockade (central or complete)

**Figure 3:**
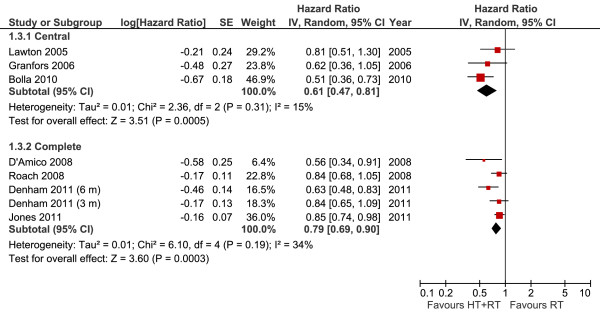
**Overall Survival According to Hormonal Blockade Meta-analysis**. Meta-analysis of overall survival, comparing central androgen deprivation (goserelin or orchiectomy) or complete hormonal blockade (LHRH analogues plus flutamide) combined with radiotherapy versus radiotherapy alone in patients with non-metastatic prostate cancer. Abbreviations: CI = confidence interval; HT--hormone therapy; IV = Generic Inverse Variance; RT--radiotherapy; SE = standard error.

The analysis of central blockade identified three studies: two with goserelin and one with orchiectomy. This subgroup analysis demonstrated that the addition of androgen suppression (defined as central blockade) led to a 39% reduction in the risk of death (HR 0.61, 95% CI 0.47-0.81, *P *= 0.0005). However, the use of complete hormonal blockade with peripheral suppressors did not result in a significant advantage (HR 0.79, 95% CI 0.69-0.90, *P *= 0.0003) (Figure 3).

#### Overall survival according to the duration of therapy

**Figure 4:**
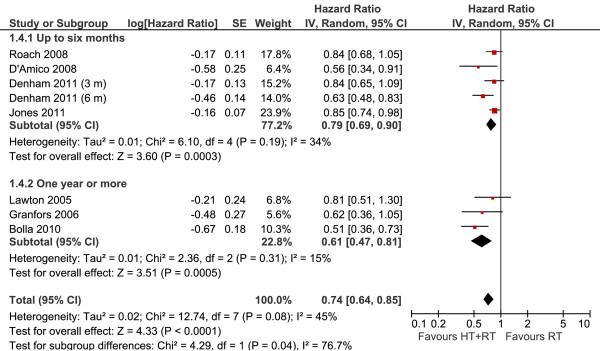
**Overall Survival According to Treatment Duration Meta-analysis**. Meta-analysis of overall survival, comparing hormone therapy combined with radiotherapy versus radiotherapy alone in patients with non-metastatic prostate cancer, according to the time of hormone treatment (up to 6 months versus one year or more). Abbreviations: CI = confidence interval; HT--hormone therapy; IV = Generic Inverse Variance; RT--radiotherapy; SE = standard error.

We divided this subgroup in two sections: studies related to treatment for up to 6 months, and studies reporting durations of 1 year or more. In both subgroups, the use of hormonal therapy was significantly better, while shorter courses of treatment demonstrated a 21% reduction in the risk of mortality (HR 0.79, 95% CI 0.69-0.90, *P *= 0.0003), longer treatment durations provided benefits of an even greater magnitude (HR 0.61, 95% CI 0.47-0.81, *P *= 0.0005) (Figure 4). The interaction test shows a statistical difference between these subgroups (*I^2 ^*= 76%; *p *= 0.04%).

### Disease-free survival

In the studies related to orchiectomy and estrogenic treatment, DFS information was unavailable. Regarding the available data, similar to the OS analysis, high heterogeneity was present for DFS evaluation. Therefore, subgroup analyses were performed to better understand the results, though statistically significant advantages were observed in all individual trials.

#### Disease-free survival per androgen suppression modality

**Figure 5:**
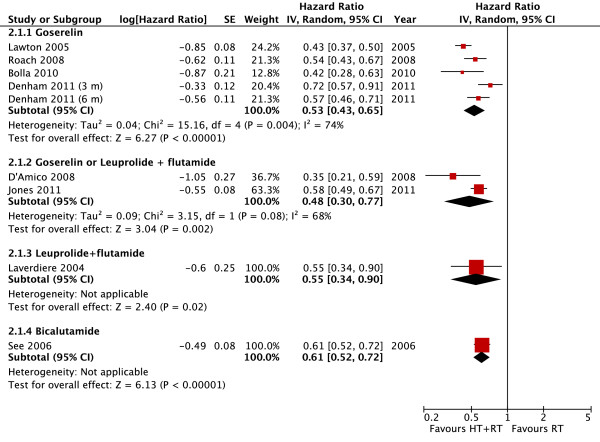
**Disease Free Survival per Androgen Suppression Meta-analysis**. Meta-analysis of disease-free survival, comparing hormone therapy combined with radiotherapy versus radiotherapy alone in patients with non-metastatic prostate cancer. Abbreviations: CI = confidence interval; HT--hormone therapy; IV = Generic Inverse Variance; RT--radiotherapy; SE = standard error.

The use of goserelin demonstrated an absolute reduction in recurrence of 47% (HR 0.53, 95% CI 0.43-0.65, *P *< 0.00001) (Figure 5). Similar to findings previously detected in the OS analysis, high heterogeneity was observed for this outcome (*I^2 ^*= 74%). The main font of heterogeneity was the shortest-term blockade arm (3 month) of Denham's study. Excluding this trial, the heterogeneity becomes acceptable (*I^2 ^*= 50%) and the reduction on recurrence associated with goserelin was more pronounced (HR 0.49, 95% CI 0.42-0.58, *P *< 0.00001).

Two studies analyzed the association of both goserelin and leuprolide to flutamide, also demonstrating benefit in DFS as well (HR 0.48, 95% CI 0.30-0.77, *P *= 0.002). One study evaluated the association of leuprolide to flutamide, with a significant benefit to patients receiving hormonal therapy (HR 0.55, 95% CI 0.34-0.90, *P *= 0.02). Additionally, an advantage to hormone therapy was observed also in the single study with bicalutamide (HR 0.61, 95% CI 0.52-0.72, *P *= 0.00001). Further details are described in Figure 5.

#### Disease-free survival according to hormonal blockade (central or complete)

**Figure 6:**
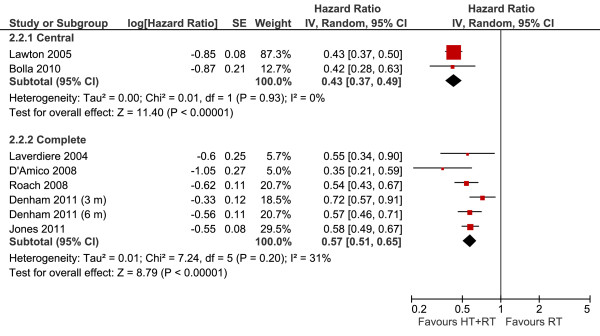
**Disease Free Survival According to Hormonal Blockade Meta-analysis**. Meta-analysis of disease-free survival, comparing androgen deprivation (goserelin or orchiectomy) or complete hormonal blockade (combination of flutamide) combined with radiotherapy versus radiotherapy alone in patients with non-metastatic prostate cancer. Abbreviations: CI = confidence interval; HT--hormone therapy; IV = Generic Inverse Variance; RT--radiotherapy; SE = standard error.

Two trials reported data regarding central blockade with goserelin (Figure 6), from which the meta-analysis showed benefit from the use of hormone therapy, reducing the risk of relapse in 57% (HR 0.43, 95% CI 0.37-0.49, *P *< 0.00001). As with complete blockade with the combination of flutamide, the gain for DFS appeared to be similar to that observed for isolated goserelin (HR 0.57, 95% CI 0.51-0.65, *P *< 0.00001).

#### Disease-free survival according to the duration of therapy

**Figure 7:**
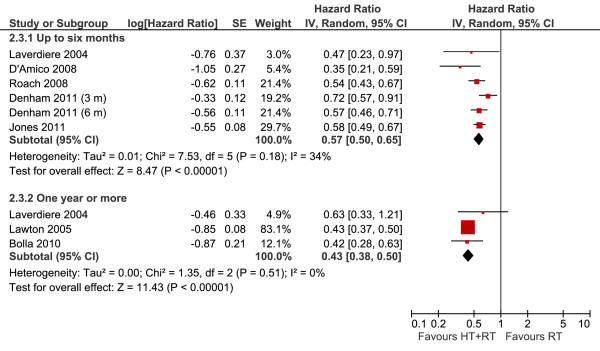
**Disease Free Survival According to Treatment Duration Meta-analysis**. Meta-analysis of disease-free survival, comparing hormone therapy combined with radiotherapy versus radiotherapy alone in patients with non-metastatic prostate cancer, according to the time of hormone treatment (up to 6 months versus 1 year or more). Abbreviations: CI = confidence interval; HT--hormone therapy; IV =Generic Inverse Variance; RT--radiotherapy; SE = standard errors.

The treatment with androgen suppression for a period not longer than 6 months (Figure 7) demonstrated the reduction of 43% in the risk of relapse (HR 0.57, 95% CI 0.50-0.65, *P *< 0.00001). Three other studies evaluated the duration of more than 1 year of therapy, with greater benefits, with a reduction of 57% in the risk of relapse (HR 0.43, 95% CI 0.38-0.50, *P *< 0.00001).

### Toxicity

Unfortunately, data extraction was compromised for this outcome as a consequence of poor reporting of adverse events among trials. Nonetheless, when trials were analysed individually, there were apparently no reports of increased toxicity.

## Discussion

An overall conclusion of this review and meta-analysis is that androgen deprivation therapy can be significantly beneficial for patients in terms of DFS and OS, when combined with radiotherapy. Nevertheless, many strategies for suppression have been studied so far, with conflicting results regarding efficacy and tolerability.

Estrogen therapy in high doses seems to be linked with higher risks of cardiovascular and thromboembolic events [24], but more importantly, failed to produce benefit in a small, single trial of 84 patients. The trials evaluating the performance of orchiectomy indicated a possible advantage to its use, though lacking statistical significance. For the reasons stated, we concluded that there is no evidence so far, to support the use of definite deprivation for such patients, nor accurate analyses of possible long-term toxicity. Hence, current studies point toward androgen suppression with LHRH analogues as more advantageous in these cases. Until the present time, goserelin was the most studied compound in this category. Further studies are warranted to demonstrate a similar benefit from other types of analogues. Additionally, combination therapy with peripheral blockade did not offer a major benefit in comparison to goserelin monotherapy.

There is still much discussion about the comparison of LHRH analogues to surgical castration regarding testosterone levels. Chemical androgen deprivation is reported to be similar among the different types of analogues used, although there is fear that sudden elevations in the level of testosterone, according to distinct pharmacodynamic characteristics, could interfere with treatment and ultimately compromise survival [25]. Authors of a recent systematic review did not provide evidence of such a presumed effect from the analogues used in practice [26].

Our study also suggested that longer androgen suppression results in better DFS and OS. Those observations are in accordance to a previous systematic review focusing the duration of deprivation therapy [27], and a randomized clinical trial comparing 6 months versus 2 years of treatment [28].

Some limiting factors in this meta-analysis must be highlighted. There were different schedules of treatment resulting in the high heterogeneity described and the distinct patient selection criteria applied from each investigator. One example is the trial from Bolla and colleagues [16], presenting the greatest reported benefit in survival with the use of 3 years of goserelin after completion of radiotherapy. Though statistically significant data were favourable for the use of such a protocol, there are still questions concerning the definition of high-risk patients in this study (T1 or T2 with histological grades greater than 3, or T3 and T4 with any grade). Distinct criteria among studies have limited the possibility to identify, with great accuracy, the groups expected to benefit from androgen suppression.

Moreover, there has been some discussion involving the type of radiotherapy performed, with the hypothesis that either conformational or intensity modulated treatments - by delivering higher doses of radiotherapy - could reduce the necessity of androgen deprivation. This idea, however, was not proven in randomised clinical trials for high-risk patients. In low-risk prostate cancer, one prospective randomised study compared conformational versus standard external beam radiotherapy, and described a reduction of biochemical relapse from 32% to 17%, without impact on overall survival [29].

Toxicity analyses were impeded by scarce reports of adverse events in the published articles. Results from observational studies have suggested the possibility that long-term androgen suppression might be related to a higher risk of metabolic syndrome [30] and osteoporosis [31], despite the lack of such descriptions in prospective work. Taking into account the tendency of higher efficacy with a longer duration of therapy, as shown in our analysis, such knowledge should be important in future trials.

## Conclusions

This meta-analysis was able to demonstrate a benefit from the combination of androgen deprivation therapy and external-beam radiotherapy for high-risk prostate cancer patients. The use of goserelin for a period longer than 1 year was associated with more beneficial survival outcomes, and should be considered standard treatment for those with higher risk of relapse. At this time, there is insufficient evidence to support other LHRH analogues or identify possible limiting long-term toxicities.

## Competing interests

This study was sponsored and partially funded by Astra-Zeneca. The sponsor had no access or interference in the preparation of the manuscript.

## Authors' contributions

ADS: conceived of the study, participated in the study design, data extraction, article selection, statistical analysis and coordination; ES: participated in the article selection, data extraction and statistical analysis; LTM: participated in the study design and manuscript preparation. AMC: participated in the study design, article selection and data extraction. All authors read and approved the final manuscript.

## Pre-publication history

The pre-publication history for this paper can be accessed here:

http://www.biomedcentral.com/1471-2407/12/54/prepub

## References

[B1] Jemal A Bray F Center MM Ferlay J Ward E Forman D Global cancer statistics CA Cancer J Clin 2011 61 2 69 90 10.3322/caac.20107 21296855

[B2] Baade PD Youlden DR Krnjacki LJ International epidemiology of prostate cancer: geographical distribution and secular trends Mol Nutr Food Res 2009 53 2 171 184 10.1002/mnfr.200700511 19101947

[B3] Bray F Lortet-Tieulent J Ferlay J Forman D Auvinen A Prostate cancer incidence and mortality trends in 37 European countries: an overview Eur J Cancer 2010 46 17 3040 3052 10.1016/j.ejca.2010.09.013 21047585

[B4] D'Amico AV Moul J Carroll PR Sun L Lubeck D Chen MH Cancer-specific mortality after surgery or radiation for patients with clinically localized prostate cancer managed during the prostate-specific antigen era J Clin Oncol 2003 21 11 2163 2172 10.1200/JCO.2003.01.075 12775742

[B5] Pisters LL The challenge of locally advanced prostate cancer Semin Oncol 1999 26 2 202 216 10597731

[B6] Hanks GE Hanlon AL Schultheiss TE Freedman GM Hunt M Pinover WH Movsas B Conformal external beam treatment of prostate cancer Urology 1997 50 1 87 92 10.1016/S0090-4295(97)00226-4 9218024

[B7] Horwitz EM Hanlon AL Hanks GE Update on the treatment of prostate cancer with external beam irradiation Prostate 1998 37 3 195 206 10.1002/(SICI)1097-0045(19981101)37:3<195::AID-PROS10>3.0.CO;2-C 9792138

[B8] Zagars GK Johnson DE von Eschenbach AC Hussey DH Adjuvant estrogen following radiation therapy for stage C adenocarcinoma of the prostate: long-term results of a prospective randomized study Int J Radiat Oncol Biol Phys 1988 14 6 1085 1091 10.1016/0360-3016(88)90383-5 3133327

[B9] Bolla M Gonzalez D Warde P Dubois JB Mirimanoff RO Storme G Bernier J Kuten A Sternberg C Gil T Improved survival in patients with locally advanced prostate cancer treated with radiotherapy and goserelin N Engl J Med 1997 337 5 295 300 10.1056/NEJM199707313370502 9233866

[B10] Parmar MK Torri V Stewart L Extracting summary statistics to perform meta-analyses of the published literature for survival endpoints Stat Med 1998 17 24 2815 2834 10.1002/(SICI)1097-0258(19981230)17:24<2815::AID-SIM110>3.0.CO;2-8 9921604

[B11] Tierney JF Stewart LA Ghersi D Burdett S Sydes MR Practical methods for incorporating summary time-to-event data into meta-analysis Trials 2007 8 16 10.1186/1745-6215-8-16 17555582 PMC1920534

[B12] Sterne JA Egger M Smith GD Systematic reviews in health care: Investigating and dealing with publication and other biases in meta-analysis BMJ 2001 323 7304 101 105 10.1136/bmj.323.7304.101 11451790 PMC1120714

[B13] DerSimonian R Laird N Meta-analysis in clinical trials Control Clin Trials 1986 7 3 177 188 10.1016/0197-2456(86)90046-2 3802833

[B14] Higgins JP Thompson SG Deeks JJ Altman DG Measuring inconsistency in meta-analyses BMJ 2003 327 7414 557 560 10.1136/bmj.327.7414.557 12958120 PMC192859

[B15] Lawton CA Winter K Grignon D Pilepich MV Androgen suppression plus radiation versus radiation alone for patients with stage D1/pathologic node-positive adenocarcinoma of the prostate: updated results based on national prospective randomized trial Radiation Therapy Oncology Group 85-31 J Clin Oncol 2005 23 4 800 807 10.1200/JCO.2005.08.141 15681524

[B16] Bolla M Van Tienhoven G Warde P Dubois JB Mirimanoff RO Storme G Bernier J Kuten A Sternberg C Billiet I External irradiation with or without long-term androgen suppression for prostate cancer with high metastatic risk: 10-year results of an EORTC randomised study Lancet Oncol 2010 11 11 1066 1073 10.1016/S1470-2045(10)70223-0 20933466

[B17] Granfors T Modig H Damber JE Tomic R Long-term followup of a randomized study of locally advanced prostate cancer treated with combined orchiectomy and external radiotherapy versus radiotherapy alone J Urol 2006 176 2 544 547 10.1016/j.juro.2006.03.092 16813885

[B18] Laverdiere J Nabid A De Bedoya LD Ebacher A Fortin A Wang CS Harel F The efficacy and sequencing of a short course of androgen suppression on freedom from biochemical failure when administered with radiation therapy for T2-T3 prostate cancer J Urol 2004 171 3 1137 1140 10.1097/01.ju.0000112979.97941.7f 14767287

[B19] Roach M Bae K Speight J Wolkov HB Rubin P Lee RJ Lawton C Valicenti R Grignon D Pilepich MV Short-term neoadjuvant androgen deprivation therapy and external-beam radiotherapy for locally advanced prostate cancer: long-term results of RTOG 8610 J Clin Oncol 2008 26 4 585 591 10.1200/JCO.2007.13.9881 18172188

[B20] D'Amico AV Chen MH Renshaw AA Loffredo B Kantoff PW Risk of prostate cancer recurrence in men treated with radiation alone or in conjunction with combined or less than combined androgen suppression therapy J Clin Oncol 2008 26 18 2979 2983 10.1200/JCO.2007.15.9699 18565884

[B21] Jones CU Hunt D McGowan DG Amin MB Chetner MP Bruner DW Leibenhaut MH Husain SM Rotman M Souhami L Radiotherapy and short-term androgen deprivation for localized prostate cancer N Engl J Med 2011 365 2 107 118 10.1056/NEJMoa1012348 21751904

[B22] Denham JW Steigler A Lamb DS Joseph D Turner S Matthews J Atkinson C North J Christie D Spry NA Short-term neoadjuvant androgen deprivation and radiotherapy for locally advanced prostate cancer: 10-year data from the TROG 96.01 randomised trial Lancet Oncol 2011 12 5 451 459 10.1016/S1470-2045(11)70063-8 21440505

[B23] See WA Tyrrell CJ The addition of bicalutamide 150 mg to radiotherapy significantly improves overall survival in men with locally advanced prostate cancer J Cancer Res Clin Oncol 2006 132 Suppl 1 S7 16 16896884 10.1007/s00432-006-0132-6 PMC12161113

[B24] Marselos M Tomatis L Diethylstilboestrol: II, pharmacology, toxicology and carcinogenicity in experimental animals Eur J Cancer 1992 29A 1 149 155 1445734 10.1016/0959-8049(93)90597-9

[B25] Boccafoschi C Prostate cancer and androgen deprivation: optimal castration? Prospects and developments Arch Ital Urol Androl 2011 83 1 63 66 21585175

[B26] Vilar Gonzalez S Maldonado Pijuan X Evidence-based medicine: comparative analysis of luteinizing hormone-releasing hormone analogues in combination with external beam radiation and surgery in the treatment of carcinoma of the prostate BJU Int 2010 107 8 1200 1208 21078049 10.1111/j.1464-410X.2010.09827.x

[B27] Cuppone F Bria E Giannarelli D Vaccaro V Milella M Nistico C Ruggeri EM Sperduti I Bracarda S Pinnaro P Impact of hormonal treatment duration in combination with radiotherapy for locally advanced prostate cancer: meta-analysis of randomized trials BMC Cancer 2010 10 675 10.1186/1471-2407-10-675 21143897 PMC3016294

[B28] Horwitz EM Bae K Hanks GE Porter A Grignon DJ Brereton HD Venkatesan V Lawton CA Rosenthal SA Sandler HM Ten-year follow-up of radiation therapy oncology group protocol 92-02: a phase III trial of the duration of elective androgen deprivation in locally advanced prostate cancer J Clin Oncol 2008 26 15 2497 2504 10.1200/JCO.2007.14.9021 18413638

[B29] Zietman AL Bae K Slater JD Shipley WU Efstathiou JA Coen JJ Bush DA Lunt M Spiegel DY Skowronski R Randomized trial comparing conventional-dose with high-dose conformal radiation therapy in early-stage adenocarcinoma of the prostate: long-term results from proton radiation oncology group/american college of radiology 95-09 J Clin Oncol 2010 28 7 1106 1111 10.1200/JCO.2009.25.8475 20124169 PMC2834463

[B30] Braga-Basaria M Dobs AS Muller DC Carducci MA John M Egan J Basaria S Metabolic syndrome in men with prostate cancer undergoing long-term androgen-deprivation therapy J Clin Oncol 2006 24 24 3979 3983 10.1200/JCO.2006.05.9741 16921050

[B31] Basaria S Lieb J Tang AM DeWeese T Carducci M Eisenberger M Dobs AS Long-term effects of androgen deprivation therapy in prostate cancer patients Clin Endocrinol (Oxf) 2002 56 6 779 786 10.1046/j.1365-2265.2002.01551.x 12072048

